# Cost-effectiveness of abatacept, tocilizumab and TNF-inhibitors compared with rituximab as second-line biologic drug in rheumatoid arthritis

**DOI:** 10.1371/journal.pone.0220142

**Published:** 2019-07-24

**Authors:** Saara Huoponen, Kalle J. Aaltonen, Jaana Viikinkoski, Jarno Rutanen, Heikki Relas, Kirsi Taimen, Kari Puolakka, Dan Nordström, Marja Blom

**Affiliations:** 1 University of Helsinki, Helsinki, Finland; 2 ESiOR Oy, Kuopio, Finland; 3 Ministry of Social Affairs and Health, Helsinki, Finland; 4 Lahti Central Hospital, Lahti, Finland; 5 Helsinki University Hospital, Helsinki, Finland; 6 Turku University Hospital, Turku, Finland; 7 South Karelia Central Hospital, Lappeenranta, Finland; Western University, CANADA

## Abstract

**Objectives:**

The objective of this study was to evaluate the cost-effectiveness of abatacept, tocilizumab, and tumor necrosis factor (TNF) inhibitors as compared with rituximab in Finnish rheumatoid arthritis patients, who have previously been treated with TNF inhibitors.

**Methods:**

A patient-level simulation model was developed to predict costs and outcomes associated with four biological drugs (abatacept, tocilizumab, rituximab and TNF inhibitors) in the treatment of rheumatoid arthritis. Following lack of efficacy or adverse events, the patients were switched to another biological drug until all four options were exhausted. After that, the patients were assumed to receive a 6^th^ line treatment until death. The patients’ baseline characteristics and regression models used in the simulation were based on observational data from the National Register for Biological Treatments in Finland. Direct costs comprised drug costs, administration costs, costs of switching, and outpatient and inpatient care, while indirect costs included disability pension and sick leaves due to rheumatoid arthritis. Several subgroup and deterministic sensitivity analyses were conducted.

**Results:**

Drug costs were the lowest for rituximab, but when administration costs and costs of switching were included, drug costs were the lowest for TNF inhibitors. Abatacept was associated with the highest drug costs, whereas rituximab was associated with the highest healthcare costs. In total, TNF inhibitors had the lowest direct costs, while rituximab had the highest direct costs. The amount of quality-adjusted life years (QALY) gained ranged from 9.405 for rituximab to 9.661 for TNF inhibitors. TNF inhibitors, abatacept, and tocilizumab were dominant in comparison to RTX.

**Conclusions:**

TNF inhibitors, abatacept, and tocilizumab had lower costs and higher QALYs than rituximab, and therefore, they were dominant in comparison to rituximab. As TNF inhibitors had the lowest costs and highest QALYs, they were the most cost-effective treatment option.

## Introduction

Rheumatoid arthritis (RA) causes significant costs for society due to the increased use of healthcare resources, sick leaves and early retirements. Consequently, effective anti-rheumatic therapies have the potential to reduce societal costs while also improving the patients’ quality of life. According to current Finnish Care Guideline, treatment of RA should be initially treated with a combination of methotrexate (MTX), hydroxychloroquine (HCQ), sulfasalazine (SSZ) and a low-dose glucocorticoid [1]. In case of an insufficient response or intolerance, biological disease modifying anti-rheumatic drugs (bDMARDs), i.e. abatacept (ABA), tocilizumab (TCZ), rituximab (RTX), sarilumab (SAR), and tumour necrosis factor (TNF) inhibitors including etanercept (ETN), adalimumab (ADA), infliximab (IFX), certolizumab pegol (CTZ), and golimumab (GOL) are prescribed [1,2]. The bDMARDs have comparable efficacy and not significantly differing safety profiles [1,3–7]. bDMARDs are recommended to be used in combination with methotrexate rather than as monotherapy due to better efficacy and reduced immunogenicity. However, ABA and TCZ as monotherapy have been shown to have similar efficacy as in combination with MTX [8,9].

A previously published systematic review indicated RTX as the most cost-effective bDMARD among patients with an insufficient response to bDMARD treatment [10]. Previous cost-effectiveness analyses were mostly based on the efficacy measured in randomized controlled trials (RCT), and might therefore, have limited generalizability to routine healthcare owing to stringent inclusion criteria and brief follow-up [11]. Nevertheless, a previous study based on observational data showed RTX to provide small savings and quality-adjusted life year (QALY) gains as a second line treatment as compared with TNF inhibitors [12]. Whether these findings are generalizable to Finnish healthcare is unknown. Also, introduction of biosimilars has lowered the treatment costs of some TNF-inhibitors as well as rituximab. The treatment of RA, especially in the field of biological drugs, has changed a lot during previous years, and therefore, there is a need for cost-effectiveness analyses based on real-world data reflecting current treatment practice and providing valuable information for health-care decision making. The objective of this study was to evaluate the cost-effectiveness of ABA, TCZ, and TNF inhibitors as compared with RTX in RA patients, who have previously been treated with TNF inhibitor using Finnish patient-level registry data.

## Materials and methods

### Model structure

**Fig 1:**
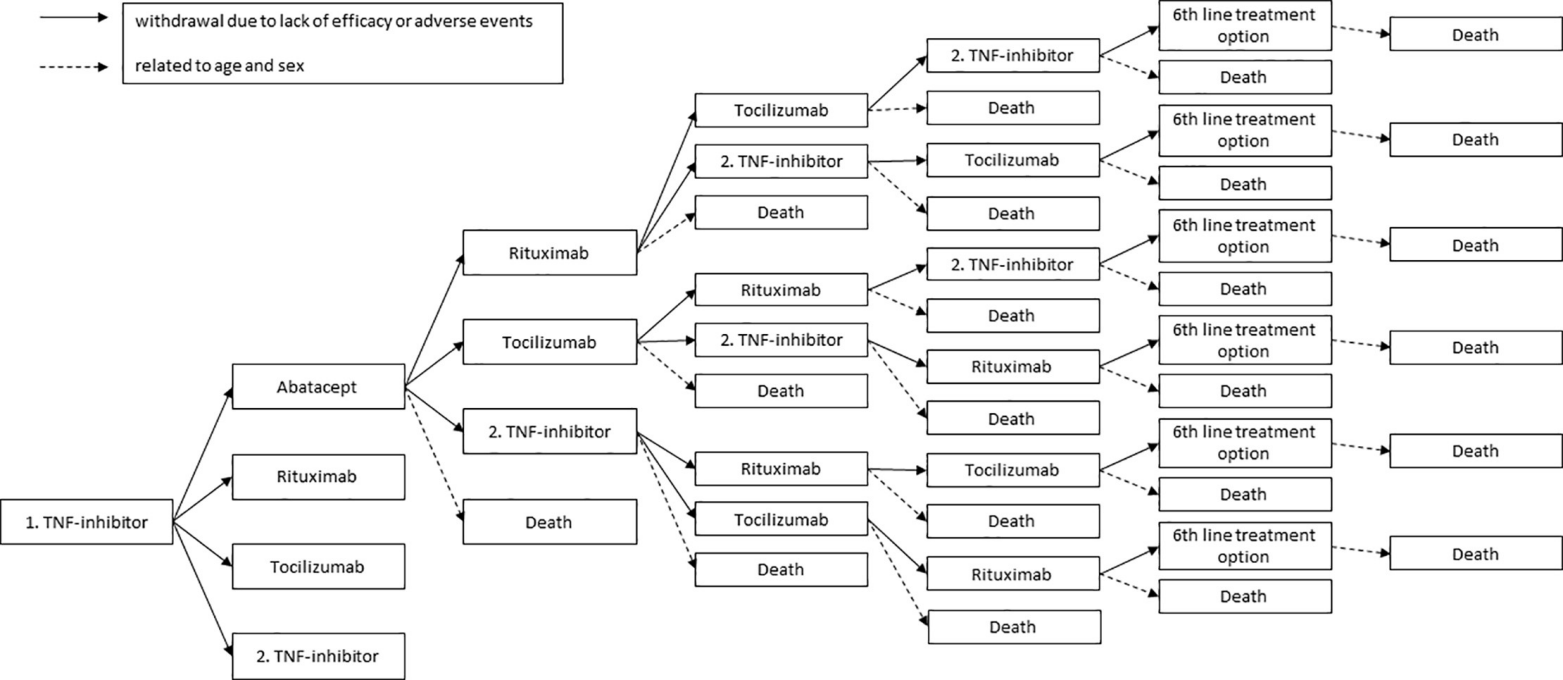
Treatment pathways included in the model. The pathways for rituximab, tocilizumab and TNF-inhibitors follow identical logic as for abatacept. DAS28 = Disease Activity Score 28, HAQ = Health Assessment Questionnaire, TNF inhibitor = tumor necrosis factor inhibitor.

We developed a patient-level simulation model using R statistical programming language 3.2.2 to estimate costs and outcomes associated with different bDMARDs in the treatment of RA. The population consisted of RA patients who had previously used a TNF inhibitor as their first bDMARD and were about to begin their second bDMARD. The model simulated four alternative treatment regimens: ABA, RTX, TCZ and a second TNF inhibitor. In this simulation TNF inhibitors were considered together as a single group rather than as individual drugs due to the same mechanism of action and similar effectiveness [11,13]. Different routes of administration for ABA and TCZ were also pooled as single groups. In the base case analysis, the choice of admin route of ABA and TCZ was based on the National Register for Biologic Treatment in Finland (ROB-FIN). Every treatment regimen was simulated by identical cohorts of 1,000 patients. In case the simulated bDMARD treatment was discontinued either due to lack of efficacy or adverse events, patients were switched to another bDMARD in the beginning of the next cycle until the patient had exhausted all four treatment options [14–16]. After that, patients were assumed to be treated with a 6^th^ line treatment until death. The outline of the model is presented in Fig 1.

Patients were assumed to remain on any given treatment for at least 6 months. At the end of each six-month period, the model individually evaluated for each person whether he or she would continue treatment, discontinue it or die. The length of the time period was set to six months as this was the average time interval for routine care visits to rheumatologists in the (ROB-FIN).

The simulation used regression models to predict outcomes and costs for each patient individually in each period (S1 Fig). Each patients’ characteristics, history of drug use and past treatment responses were recorded in the model and utilized as predictors for future outcomes and costs. The analysis was conducted from a societal perspective as the study included both direct and indirect costs. Half-cycle correction was applied to both outcomes and costs. We analyzed direct costs and both direct and indirect costs separately [17]. Health outcomes also were expressed as QALYs. Based on the Finnish recommendations for health economic evaluations, all costs and benefits were discounted at 3.0% annually [17]. Primary outcome of the simulation was incremental cost-effectiveness ratio (ICER) per QALY including only direct costs.

### Data sources

Primary data source for the model was the ROB-FIN and all assumptions were based on observed data unless otherwise mentioned. ROB-FIN has been described in previous publications [11,18–20]. In brief, ROB-FIN is a longitudinal observational cohort study established in 1999 to monitor effectiveness and safety of biologic drugs in treatment of RA and was originally based on structured data collection forms submitted by rheumatologists on patients´ routine care visits to outpatient specialized healthcare. Starting in 2007, most of the data have been retrieved from electronic patient monitoring systems. Additional data on the patients’ hospitalization and outpatient visits as well as sick leaves and disability pensions were acquired from national healthcare registers, which were linked to ROB-FIN using social security numbers.

Observed ROB-FIN data from baseline visit as well as subsequent follow-up visits were used to construct the regression models later used to predict patients’ treatment response, utility and costs in the simulation. Independent variable selection for the regression models was based on Akaike Information Criteria although the choice of bDMARD therapy and its interactions with other variables were always included where appropriate. Missing data among disease activity parameters were imputed by multiple imputation whereas information on treatments and use of healthcare resources were considered complete. Patients lost to follow-up were treated as uninformative censoring while information on treatment discontinuations were utilized in the modeling.

### Model inputs

#### Baseline characteristics

The model population were sampled with replacement among the patients included in ROB-FIN about to start their second bDMARD therapy thus preserving any potential correlation between the variables. The baseline variables included in the model comprised age, sex, weight, Body Mass Index (BMI), Health Assessment Questionnaire (HAQ), DAS28, time from diagnosis of rheumatic disease, Rheumatoid Factor (RF) status, and concomitant use of MTX, SSZ and HCQ along with the patients’ healthcare costs during the past 12 months. The patients´ weight, BMI and RF status were fixed at the baseline values.

#### Clinical effectiveness

Treatment effectiveness was defined as an achievement of at least American College of Rheumatology (ACR) 20% improvement, a moderate European League Against Rheumatism (EULAR) response, or a Disease Activity Score 28 (DAS28) value of less than 3.2 [14–16]. The actual observed data were used to construct regression models, which in turn were used to predict the responses in the simulation. Based on expert opinion, the treatment response to the 6^th^ line treatment options was assumed to be the same as averaged treatment response to all biological treatments in the model.

The patients’ underlying HAQ score was assumed to remain fixed at the baseline value. The change in HAQ score in comparison to baseline was modeled and subsequently predicted using a linear regression model. Any effect the treatment could have on these disease activity parameters was assumed to be temporary and re-evaluated in the next period.

#### Clinical safety

In addition to lack of efficacy, treatment might be discontinued due to adverse events and other reasons. The risk of discontinuation due to adverse events and other reasons besides lack of efficacy was observed to be 0.068 across all bDMARDs in average based on the available data in ROB-FIN. After every six-month time period 0.017, 0.010 and 0.009 per cent of MTX, SSZ and HCQ users discontinued the use of the said co-treatment, respectively.

#### Mortality

Mortality rate adjusted by age and sex for patient-level data was based on the life table in 2017 published by Statistics Finland [21]. In this model, RA was associated with mortality of general population [22–24]. Despite using lifetime horizon in the model, we assumed that patients would die at the latest at the age of 100 due to lack of mortality data beyond that age.

#### Utility

Quality-adjusted life years (QALY) were calculated corresponding to EuroQol five dimensions questionnaire (EQ-5D-3L) utilities predicted from the HAQ scores utilizing a multinomial logistic regression model. The regression model was based on data from a survey including both HAQ and EQ-5D-3L conducted in Finland in 2009 [20]. Obtained EQ-5D-3L health stages were valued with the Finnish tariff [25].

#### Costs

Direct costs comprised drug costs, administration costs of infusions, costs of switching, outpatient and inpatient care, while indirect costs included early retirement due to RA and sick leave. Costs of drugs were based on the Finnish price list including the retail price without value added tax of drugs and the dose in the label [26]. The costs for infusion drugs were the wholesale prices of Helsinki and Uusimaa Hospital District (Aaltonen T, personal communication, April 20, 2016). The prices of biosimilar IFX, ETN, and ADA were used in the base case analysis, whereas the price of biosimilar of RTX was taken into account in sensitivity analysis. Administration costs of infusions including intravenous treatment cost prices of bDMARDs for RA at Finnish hospitals were derived from the Finnish study by Soini et al [27]. The price for TNF inhibitor group was an average for individual TNF inhibitors weighed by their actual usage. Similarly, the costs for ABA and TCZ were weighed averages based on the prices of subcutaneously and intravenously administered products. In the base case analysis, the bDMARD was subcutaneously administered for 15 and 7 per cent of ABA and TCZ users, respectively. Drug costs, administration costs of infusions and dosages are presented in S1 Table. Cost of switching was assumed to be equal to the cost of one healthcare visit to internal medicine specialist in specialized outpatient healthcare [28]. Based on the expert opinion and in line with their effectiveness, the costs of the 6^th^ line treatment option were considered to be same as the averaged costs of currently available biological treatments.

Patients’ biannual healthcare and indirect costs were modeled using a linear regression model based on actual observed data and later predicted in the simulation using this model. Drugs in outpatient care were in 2019 euros and all other costs were converted to 2017 euros using the price indices of Statistics Finland [29].

### Sensitivity analyses

The model was stochastic in nature and the 1000 unique model runs with different seeds for random number generator quantified the variability in the results. Several subgroup and deterministic sensitivity analyses were carried out based on 300 model runs to explore uncertainty and heterogeneity of the model results. As ABA and TCZ can be administered either subcutaneously or intravenously, we evaluated the influence of the choice of the administration route on the results in the deterministic sensitivity analyses. Even though HAQ progression was associated with age and the number of biologic treatment in the base case analysis, we used annual HAQ progression rates of 0.03 and 0.06 in the sensitivity analyses [30]. Also, we included a scenario where we employed a British tariff to value EQ-5D health stages [31]. Although the patent of RTX has expired, RTX biosimilar approved for the treatment of RA is not on the market in Finland. Therefore, we considered RTX biosimilar with a price discount of 30% in comparison to the reference medicinal product in sensitivity analysis. The time horizon of 10 years and the discounting rate of 0% and 6% were explored in the sensitivity analyses [17]. In subgroup analyses we evaluated the heterogeneity of the results related to the use of the concomitant csDMARD therapy, age, body mass index (BMI), gender, presence of RF, prior use of MTX, and primary response to the first TNF inhibitor.

### Ethical approval

Ethical approval for this study was granted by the Helsinki University Central Hospital ethical committee (73/13/03/00/2014). The study permit to use the patient records and cost data was granted by the Finnish National Institute for Health and Welfare (THL/1497/.5.05.00/2013), Finnish Population Registry (262/410/16) and the Social Insurance Institution of Finland (Kela 7/522/2016). Written informed patient consents were acquired from patients who had been included in ROB-FIN prior to the introduction of the electronic patient monitoring systems.

## Results

### Baseline characteristics

At baseline, median age of patients included in the study was 56 years (Table 1). Most of the patients were female. Median HAQ score was 1.1, whereas median DAS28 was 4.6. More than half of the patients had a treatment response to the first TNF inhibitor. The characteristics of the patients at the baseline are presented in Table 1.

**Table 1:** Characteristics of the patients at the baseline.

Variable	Median / %	The first and the third quartile / n
Age (years)	56	47–63
Male	25	361
Weight	72	65–82
BMI (kg/m2)	26	24–28
DAS28	4.6	3.5–5.7
HAQ	1.1	0.6–1.6
Methotrexate	55	786
Hydroxychloroquine	27	384
Sulfasalazine	18	257
Patients with positive rheumatoid factor status	92	1,306
Patients with response to the first TNF inhibitor	65	922
Time from the diagnosis of rheumatoid arthritis (years)	13	7.3–20
Outpatient and inpatient care costs during the last 6 months (€)	1,737	890–4,561
Outpatient and inpatient care costs during the last 12 months (€)	3,542	1,601–7,723

BMI = body mass index, DAS28 = Disease Activity Score 28, HAQ = Health Assessment Questionnaire, TNF inhibitor = tumor necrosis factor inhibitor

### Base-case analysis

Lifetime drug costs without administration costs and costs of switching were the lowest for RTX, but when administration costs and costs of switching were included, drug costs were the lowest for TNF inhibitors (Table 2). ABA had the highest drug costs. However, ABA had the lowest healthcare costs, while RTX had the highest healthcare costs. In total, the lowest and highest direct costs were associated with the TNF inhibitors and RTX, respectively. Indirect costs ranged from 148,718 € for TNF inhibitors to 165,300 € for RTX. Drug costs including administration costs and costs of switching represented over half of the total costs. QALYs ranged from 9.405 of RTX to 9.661 of TNF inhibitors. In our model, patients died in average at the age of 85.59 (standard deviation 9.96).

**Table 2:** Lifetime costs and QALYs of bDMARDs per patient as a second-line biologic therapy in rheumatoid arthritis. All costs and QALYs were discounted at 3% per year.

	Abatacept	Tocilizumab	TNF inhibitors	Rituximab
Drug costs, €	211,384	211,071	201,436	201,407
Administration costs, €	57,799	56,983	47,656	48,908
Drug costs including administration costs and costs of switching, €	270,028	268,928	249,937	251,291
Outpatient and inpatient costs, €	25,805	32,749	26,968	69,681
Direct costs, €	295,833	301,677	276,905	320,972
Indirect costs, €	154,293	159,480	148,718	165,300
Total costs, €	450,126	461,157	425,623	486,272
Life years	19.271	19.271	19.272	19.272
QALYs	9.475	9.407	9.661	9.405

bDMARD = biological disease-modifying anti-rheumatic drug, QALY = Quality-adjusted life year, TNF inhibitor = tumor necrosis factor inhibitor

**Fig 2:**
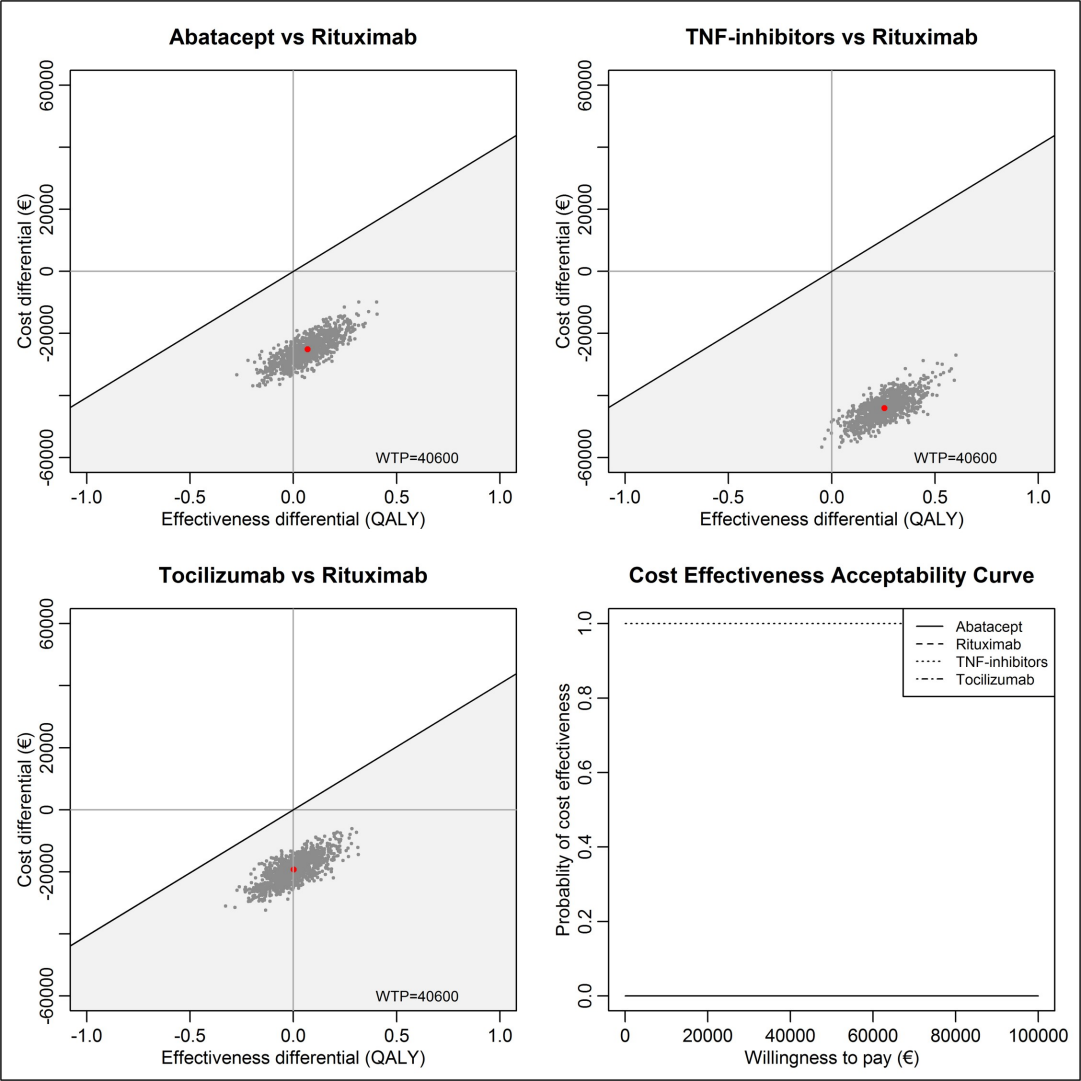
Incremental cost-effectiveness planes and cost-effectiveness acceptability curve for abatacept, TNF inhibitors, and tocilizumab in comparison to rituximab for the treatment of rheumatoid arthritis as a second-line therapy in a lifetime scenario. ICER = incremental cost-effectiveness ratio, QALY = quality-adjusted life year, TNF-inhibitors = tumor necrosis factor inhibitors.

TNF inhibitors, ABA, and TCZ had lower costs and higher QALYs than RTX, and therefore, they were dominant in comparison to RTX (Table 3 and Fig 2). TNF inhibitors are the most cost-effective treatment option, as they have the lowest costs and the highest lifetime QALYs.

**Table 3:** Lifetime cost-effectiveness of bDMARDS in comparison to rituximab as a second-line biologic therapy in rheumatoid arthritis. All costs and QALYs were discounted at 3% per year.

	Abatacept vs. rituximab	Tocilizumab vs. rituximab	TNF inhibitors vs. rituximab
Incremental costs, € (only direct costs)	-25,139	-19,295	-44,067
Incremental costs, € (both direct and indirect costs)	-36,145	-25,115	-60,649
Incremental life years	-0.00090	-0.00059	-0.00006
Incremental QALYs	0.070	0.002	0.256
ICER, €/QALY (only direct costs)	Dominant	Dominant	Dominant
ICER, €/QALY (both direct and indirect costs)	Dominant	Dominant	Dominant

bDMARD = biological disease-modifying anti-rheumatic drug, ICER = Incremental cost-effectiveness ratio, QALY = Quality-adjusted life year, TNF inhibitor = tumor necrosis factor inhibitor

### Sensitivity analysis

The results regarding costs and QALYs from the subgroup and deterministic sensitivity analysis are presented in S2 and S3 Tables. Instead of intravenous administration, the use of self-administered ABA and TCZ decreased the administration costs of drugs, but the choice of route of administration had no effect on other costs. Similarly to base case results, RTX had the highest costs and lowest QALYs when they were discounted at 0%, but when the discounting rate of 6% was used, TCZ had the lowest QALYs. When the time horizon of 10 years was used, RTX had the lowest drug costs including the costs of switching and the administration costs, while TCZ was associated with the lowest QALYs. As compared with RTX, TNF inhibitors were dominant, whereas the incremental cost-effectiveness ratio for ABA was 144,213 €/QALY when the time horizon of 10 years was used. RTX as a second-line treatment option had the highest direct costs even when the price discount of 30% for RTX biosimilar was used, owing to the highest outpatient and inpatient costs for RTX. Indirect costs increased and QALYs decreased in case the bi-annual HAQ progression was fixed at 0.03 or 0.06. Removing half-cycle correction had little effect on the results. QALYs decreased when the health stages were valued with the British tariff. The costs were higher and QALYs were lower for women than for men. As compared with patients with no response to the first TNF inhibitor, primary responders had slightly higher costs and QALYs. The QALYs ranged from 11.960 to 12.370 for patients with negative RF status, while QALYs ranged from 9.159 to 9.415 for patients with positive RF status. Also the costs were higher among patients with negative RF status as compared to RF-positive patients. Furthermore, concomitant use of MTX or other non-biologic therapies lead to increased QALYs in comparison to non-use of MTX or biologic monotherapy, respectively.

## Discussion

Based on our patient-level simulation model using real-world data from Finland, TNF inhibitors, ABA, and TCZ as a second-line biologic treatments for RA were dominant as compared with RTX. Even though a commonly referred threshold for cost-effectiveness has not been published in Finland, we have used the willingness to pay threshold of 40,600€, representing the Finland´s gross domestic product per capita in 2017 per QALY gained in this study. [32] According to our model, RTX was associated with the lowest drug costs, but when administration costs and costs of switching were included, TNF inhibitors had the lowest drug costs. RTX had the highest healthcare costs, and in total, RTX was associated with the highest drug costs. TCZ was associated with the lowest effectiveness. TNF inhibitors had the lowest costs and the highest QALYs, and therefore, they were the most cost-effective treatment option.

Consistent with our results, previous studies showed similar effectiveness between bDMARDs in patients with RA failing on TNF inhibitor [33,34], whereas other studies suggest that changing to RTX is more effective than switching to an alternative TNF inhibitor [35,36]. Unlike our results, previously published results by Lindgren et al. found that RTX treatment was associated with the lowest overall costs and was the most effective option [12]. Similarly, in a head-to-head RCT between RTX, TNF-inhibitors, ABA and TCZ, RTX was associated with both the lowest costs and the highest QALY gain [37]. The Finnish study reported also RTX to be most cost-effective treatment alternative for patients with RA who have failed TNF inhibitor treatment [38]. According to this study, life years were highly similar between all bDMARDs, but they were the highest for RTX.

The main advantage of this study was that real-world data on costs and effectiveness were based on registry data from the same population. Baseline characteristics of the model and population and the assumptions used in the model were mainly based on ROB-FIN data representing routine clinical practice. Compared to the previous studies mostly based on efficacy derived from RCTs, our simulation was based on observational data and therefore results are likely to be more generalizable to the Finnish healthcare setting [11]. In addition, cost data derived from comprehensive Finnish national registers were employed whenever possible. To eliminate the bias caused by confounders in the observational data, we used several regression models to predict the patients’ treatment response, utility and costs in the simulation.

Our model memorized changes in patient characteristics over time and enabled simulation based on individual patients’ history. The history of what had happened was an important aspect of the model because it affected the occurrence of future events, their consequences and valuations, and many other aspects of the simulation. The simulation followed up patients for their complete lifetime, because RA is a chronic disorder that progresses over time. As such, long-term consequences of any differences in disease progression, effect on life expectancy, or drug discontinuation rates were assessed. The first TNF inhibitor was not included in this model as it was assumed to be identical between the comparators and would not affect the results. Reasons for discontinuation of RTX treatment were severely underreported in ROB-FIN data and therefore, the risk of adverse events was assumed to be equal across all treatment regimens. Also, the discontinuation probabilities for non-biologic co-therapies were assumed to be similar between the users of different biologics.

Because of high costs of original bDMARDs, interest has grown in biosimilars that are comparable to the reference medicinal product in terms of efficacy and safety. Many original biologic drugs have reached, or are approaching, patent expiry. This will lead to increasing development and use of biosimilar drugs in the future, offering considerable savings in comparison with the reference medicinal product. Price competition after patent expiry may also reduce the price of reference medicinal product, leading to remarkable cost savings. Biosimilars of ETN, IFX, and ADA were considered in this analysis. The price of RTX biosimilar used for the treatment of RA was not available, but we used price discount of 30% for RTX biosimilar in the sensitivity analysis based on the Finnish legislation concerning the confirming a reasonable wholesale price for medicinal products [39]. However, the price discount may be even bigger in the future.

A potential limitation of this study is related to the dosing interval of RTX. As compared with other biologics, RTX has a unique mode of action and long dosing interval. In maintenance treatment, RTX infusions were often administered on demand in Finland. Consequently, the patients treated with RTX came to visit the rheumatologist only after the effect of the previous infusion began to wear off, which is very likely to lead to an underestimation of the effectiveness of RTX in our data, and therefore, leading to overestimation of healthcare costs. Based on the registry data used in this study, RTX was administered every 7.98 months in maintenance treatment. Similar dosing interval of RTX was reported by Keystone et al [40], whereas mean dosing interval of RTX varied between 11 and 13 months in daily clinical practice in Finland according to the study by Valleala et al [41]. Therefore, a relatively short dosing interval used in this study might also be one reason to cause high costs of RTX treatment. In this model RA was associated with mortality of general population, which is another limitation of this study. According to the study by Kroot et al, mortality of RA patients was comparable with the expected mortality of the general population of the Netherland up to 10 years of RA [23]. This finding was in line with the study by Lindgqvist and Eberhardt et al. [24]. Furthermore, Lacaille et al found that mortality gap between RA and the general population in the first five years was not observed in people with RA onset after year 2000 [22]. However, it is notable that the patient populations in these studies comprised of patients with recent onset of RA. Although mortality has decreased among RA patients over the past decades, the general belief is that patients with RA, especially the more severe cases, have a shortened life expectancy compared with the general population [42]. We assume that differences in QALYs between bDMARDS would have been bigger, but the order of these results would have been the same, if the association between disease activity and mortality in RA patients had been considered in the model. We did not however, have sufficient data to create a prediction model for mortality, which can be considered a limitation. Furthermore, patients’ erosive progression could not be taken into account as data on this subject was not available in ROB-FIN. The effect could be mitigated by inclusion of HAQ scores, which have been shown to be correlated with the presence of joint erosions [43]. Some bias could also be caused by the lack of a generic health related quality of life instrument. When we employed another valuation method in sensitivity analysis, the QALYs decreased when health stages were valued with the British tariff.

## Conclusion

Our patient-level simulation based on observational data showed that TNF inhibitors were associated with the lowest costs and highest QALYs, whereas RTX had the highest costs and lowest QALYs. TNF inhibitors, ABA, and TCZ were dominant in comparison to RTX. As TNF inhibitors had the lowest costs and highest QALYs, they were the most cost-effective treatment option.

## Supporting information

S1 Fig Influence diagram for a patient-level simulation model of cost-effectiveness of biologics for the treatment of rheumatoid arthritis. ACR = American College of Rheumatology response, bDMARD = biological disease-modifying anti-rheumatic drugs DAS28 = Disease Activity Score 28, EULAR = European League Against Rheumatism response, HAQ = Health Assessment Questionnaire, HCQ = hydroxychloroguine, MTX = methotrexate, SSZ = sulfasalazine.
(TIF)

S1 Table Drug costs, administration costs of infusions, and dosages of biological drugs in the treatment of rheumatoid arthritis. iv = intravenous, sc = subcutaneous.
(DOCX)

S2 Table Results of the deterministic sensitivity analyses. ABA = abatacept, bDMARD = biological disease-modifying anti-rheumatic drug, EQ-5D-3L = EuroQol five dimensions questionnaire, ETN = etanercept, HAQ = Health Assessment Questionnaire, QALY = quality-adjusted life year, QoL = quality of life, RA = rheumatoid arthritis, TNF inhibitor = tumor necrosis factor inhibitor, TCZ = tocilizumab.
(DOCX)

S3 Table Results of the subgroup analyses. ABA = abatacept, bDMARD = biological disease-modifying anti-rheumatic drug, csDMARD, conventional synthetic disease modifying anti-rheumatic drug, ETN = etanercept, QALY = quality-adjusted life year, RA = rheumatoid arthritis, TNF inhibitor = tumor necrosis factor inhibitor, TCZ = tocilizumab.
(DOCX)
