# Supplementary figures and images for: Cost-effectiveness of abatacept, tocilizumab and TNF-inhibitors compared with rituximab as second-line biologic drug in rheumatoid arthritis
Source: PLoS One. 2019 Jul 24;14(7):e0220142. doi: 10.1371/journal.pone.0220142 (PMC6656352; doi:10.1371/journal.pone.0220142)

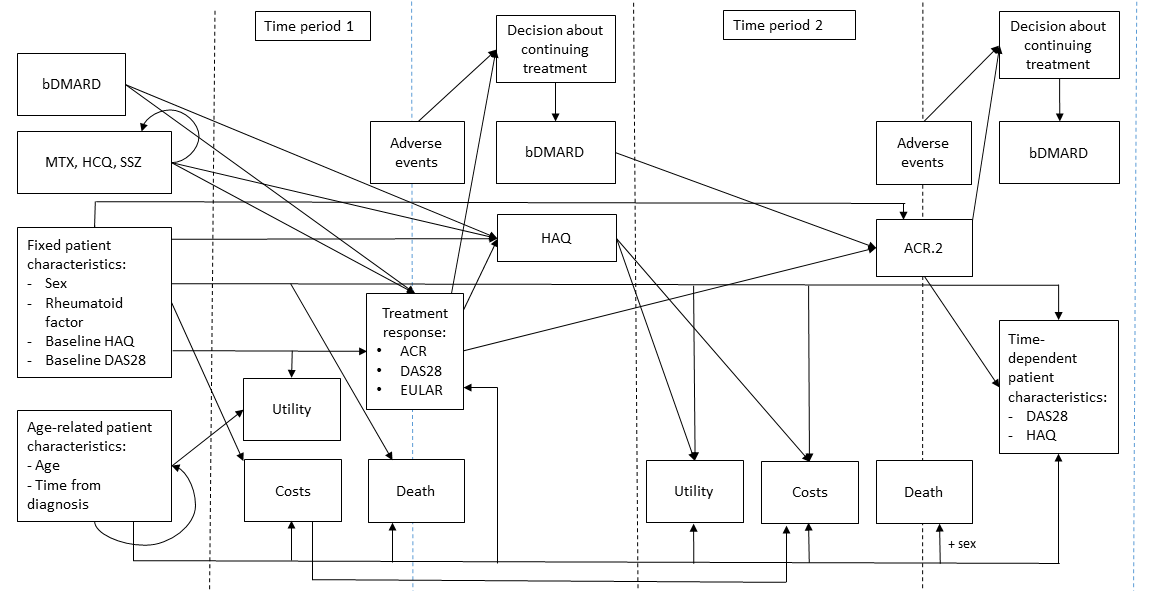

Supplement: S1 Fig — ACR = American College of Rheumatology response, bDMARD = biological disease-modifying anti-rheumatic drugs DAS28 = Disease Activity Score 28, EULAR = European League Against Rheumatism response, HAQ = Health Assessment Questionnaire, HCQ = hydroxychloroguine, MTX = methotrexate, SSZ = sulfasalazine. (TIF) [file pone.0220142.s001.tif]
