# Supplementary material for: Cost-effectiveness of abatacept, tocilizumab and TNF-inhibitors compared with rituximab as second-line biologic drug in rheumatoid arthritis
Source: PLoS One. 2019 Jul 24;14(7):e0220142. doi: 10.1371/journal.pone.0220142 (PMC6656352; doi:10.1371/journal.pone.0220142)
Supplement: S1 Table — iv = intravenous, sc = subcutaneous. (DOCX) [file pone.0220142.s002.docx]

| **Biological drug** | **Strength** | **Pharma-ceutical form** | **Route of adminis-tration** | **Pack size** | **Usual adult dose** | **Cost*/**  **package** [26]**, €** | **Cost/**  **adminis-tration** [27,29]**, €** |
| --- | --- | --- | --- | --- | --- | --- | --- |
| Abatacept | 250 mg | powder for concentrate for solution for infusion | iv | 1 vial | 500-1000 mg at weeks 0, 2, 4 , then every 4 weeks (doses according to body weight: <60 kg = 500 mg; 60-100 kg = 750 mg; >100 kg = 1000 mg) | 368.65 | 479.76 |
| Abatacept | 125 mg | solution for injection | sc | 4 pre-filled pens | 125 mg weekly | 1,067.02 | - |
| Adalimumab | 40 mg | solution for injection | sc | 2 pre-filled pens | 40 mg every two weeks | 749.37 | - |
| Certolizumab pegol | 200 mg | solution for injection | sc | 2 pre-filled pens | 400 mg at weeks 0, 2, 4, then 200mg every two weeks | 966.13 | - |
| Etanercept | 50 mg | solution for injection | sc | 4 pre-filled pens | 50 mg once weekly | 657.79 | - |
| Golimumab | 50 mg | solution for injection | sc | 1 pre-filled pen | 50 mg once a month (100mg once a month for body weight > 100 kg) | 1,086.93 | - |
| Infliximab | 100 mg | powder for concentrate for solution for infusion | iv | 1 vial | 3 mg/kg at weeks 0, 2, 6, then every 8 weeks | 148.65 | 348.42 |
| Rituximab | 500 mg | concentrate for solution for infusion | iv | 1 vial | 1000 mg at week 0 and 2, then 1000 mg every 7.98 months | 1,390.93 | 609.06 |
| Tocilizumab | 200 mg | concentrate for solution for infusion | iv | 1 vial | 8 mg/kg every 4 weeks | 344.86 | 436.62 |
| Tocilizumab | 162 mg | solution for injection | sc | 4 pre-filled pens | 162 mg once weekly | 1,067.14 | - |

* retail price excluding value added tax (VAT)
