# Supplementary material for: Cost-effectiveness of abatacept, tocilizumab and TNF-inhibitors compared with rituximab as second-line biologic drug in rheumatoid arthritis
Source: PLoS One. 2019 Jul 24;14(7):e0220142. doi: 10.1371/journal.pone.0220142 (PMC6656352; doi:10.1371/journal.pone.0220142)
Supplement: S2 Table — ABA = abatacept, bDMARD = biological disease-modifying anti-rheumatic drug, EQ-5D-3L = EuroQol five dimensions questionnaire, ETN = etanercept, HAQ = Health Assessment Questionnaire, QALY = quality-adjusted life year, QoL = quality of life, RA = rheumatoid arthritis, TNF inhibitor = tumor necrosis factor inhibitor, TCZ = tocilizumab. (DOCX) [file pone.0220142.s003.docx]

S2 Table. Results of the deterministic sensitivity analyses.

|  | Drug costs including administration costs and costs of switching, € | Outpatient and inpatient costs, € | Direct costs, € | Indirect costs, € | QALYs |
| --- | --- | --- | --- | --- | --- |
| ABA and TCZ administered subcutaneously | | | | | |
| ABA | 226,862 | 25,806 | 252,668 | 154,082 | 9.467 |
| TCZ | 228,245 | 32,761 | 261,006 | 159,247 | 9.402 |
| TNF inhibitors | 217,273 | 26,953 | 244,226 | 148,485 | 9.660 |
| RTX | 223,505 | 69,570 | 293,075 | 165,023 | 9.402 |
| ABA and TCZ administered intravenously | | | | | |
| ABA | 276,094 | 25,806 | 301,900 | 154,082 | 9.467 |
| TCZ | 273,776 | 32,761 | 306,537 | 159,247 | 9.402 |
| TNF inhibitors | 254,191 | 26,953 | 281,144 | 148,485 | 9.660 |
| RTX | 254,915 | 69,570 | 324,485 | 165,023 | 9.402 |
| Time horizon of 10 years | | | | | |
| ABA | 131,155 | 17,042 | 148,196 | 100,387 | 4.255 |
| TCZ | 128,895 | 20,491 | 149,385 | 105,570 | 4.140 |
| TNF inhibitors | 99,997 | 17,077 | 117,074 | 95,464 | 4.355 |
| RTX | 98,824 | 36,424 | 135,248 | 106,557 | 4.208 |
| Discounted at 0 % | | | | | |
| ABA | 418,805 | 34,906 | 453,710 | 202,032 | 14.926 |
| TCZ | 418,633 | 45,430 | 464,064 | 207,619 | 14.902 |
| TNF inhibitors | 406,891 | 37,190 | 444,081 | 195,605 | 15.224 |
| RTX | 410,171 | 102,581 | 512,752 | 216,918 | 14.843 |
| Discounted at 6 % | | | | | |
| ABA | 196,296 | 20,992 | 217,287 | 124,456 | 6.759 |
| TCZ | 194,816 | 26,103 | 220,919 | 129,183 | 6.681 |
| TNF inhibitors | 173,132 | 21,605 | 194,737 | 119,430 | 6.903 |
| RTX | 173,457 | 52,220 | 225,677 | 132,816 | 6.706 |
| Annual HAQ progression 0.03 | | | | | |
| ABA | 269,963 | 26,434 | 296,397 | 162,887 | 8.949 |
| TCZ | 268,938 | 33,648 | 302,586 | 168,016 | 8.859 |
| TNF inhibitors | 249,563 | 27,591 | 277,153 | 157,256 | 9.116 |
| RTX | 251,230 | 71,729 | 322,959 | 172,926 | 8.865 |
| Annual HAQ progression 0.06 | | | | | |
| ABA | 269,944 | 26,966 | 296,910 | 171,419 | 8.493 |
| TCZ | 268,823 | 34,357 | 303,180 | 176,362 | 8.388 |
| TNF inhibitors | 249,809 | 28,197 | 278,006 | 165,781 | 8.672 |
| RTX | 251,259 | 73,484 | 324,743 | 180,370 | 8.396 |
| No half-cycle correction | | | | | |
| ABA | 273,720 | 30,545 | 304,264 | 157,056 | 9.532 |
| TCZ | 271,869 | 37,437 | 309,306 | 162,206 | 9.464 |
| TNF inhibitors | 250,641 | 31,698 | 282,339 | 151,520 | 9.716 |
| RTX | 253,010 | 74,097 | 327,107 | 167,981 | 9.463 |
| Health stages valued with the British tariff [31] | | | | | |
| ABA | 269,769 | 25,806 | 295,575 | 154,082 | 7.722 |
| TCZ | 251,107 | 32,761 | 301,451 | 159,247 | 7.663 |
| TNF inhibitors | 249,753 | 26,953 | 276,706 | 148,485 | 7.883 |
| RTX | 251,107 | 69,570 | 320,678 | 165,023 | 7.667 |
| Price discount of 30 % for RTX biosimilar | | | | | |
| ABA | 268,339 | 25,806 | 294,145 | 154,082 | 9.467 |
| TCZ | 266,171 | 32,761 | 298,931 | 159,247 | 9.402 |
| TNF inhibitors | 246,449 | 26,953 | 273,402 | 148,485 | 9.660 |
| RTX | 246,040 | 69,570 | 315,610 | 165,023 | 9.402 |

ABA = abatacept, bDMARD = biological disease-modifying anti-rheumatic drug, EQ-5D-3L = EuroQol five dimensions questionnaire, ETN = etanercept, HAQ = Health Assessment Questionnaire, QALY = quality-adjusted life year, QoL = quality of life, RA = rheumatoid arthritis, TNF inhibitor = tumor necrosis factor inhibitor, TCZ = tocilizumab
