# Supplementary material for: Cost-effectiveness of abatacept, tocilizumab and TNF-inhibitors compared with rituximab as second-line biologic drug in rheumatoid arthritis
Source: PLoS One. 2019 Jul 24;14(7):e0220142. doi: 10.1371/journal.pone.0220142 (PMC6656352; doi:10.1371/journal.pone.0220142)
Supplement: S3 Table — ABA = abatacept, bDMARD = biological disease-modifying anti-rheumatic drug, csDMARD, conventional synthetic disease modifying anti-rheumatic drug, ETN = etanercept, QALY = quality-adjusted life year, RA = rheumatoid arthritis, TNF inhibitor = tumor necrosis factor inhibitor, TCZ = tocilizumab. (DOCX) [file pone.0220142.s004.docx]

S3 Table. Results of the subgroup analyses.

|  | Drug costs including administration costs and costs of switching, € | Outpatient and inpatient costs, € | Direct costs, € | Indirect costs, € | QALYs |
| --- | --- | --- | --- | --- | --- |
| Age at baseline < 50 | | | | | |
| ABA | 334,630 | 34,130 | 368,760 | 248,763 | 12.262 |
| TCZ | 334,059 | 43,249 | 377,307 | 256,036 | 12.195 |
| TNF inhibitors | 317,678 | 35,891 | 353,569 | 240,700 | 12.495 |
| RTX | 319,741 | 93,950 | 413,691 | 281,477 | 12.098 |
| Age at baseline ≥ 50 | | | | | |
| ABA | 240,290 | 21,954 | 262,244 | 110,968 | 8.185 |
| TCZ | 238,895 | 27,873 | 266,768 | 114,974 | 8.159 |
| TNF inhibitors | 218,573 | 22,874 | 241,446 | 106,390 | 8.345 |
| RTX | 219,767 | 58,501 | 278,268 | 111,807 | 8.159 |
| Body mass index (BMI) < 25 | | | | | |
| ABA | 260,899 | 26,120 | 287,020 | 146,407 | 9.810 |
| TCZ | 258,870 | 33,655 | 292,526 | 153,159 | 9.712 |
| TNF inhibitors | 242,742 | 27,491 | 270,233 | 142,423 | 9.974 |
| RTX | 244,000 | 74,371 | 318,370 | 161,850 | 9.678 |
| Body mass index (BMI) ≥ 25 | | | | | |
| ABA | 275,799 | 25,570 | 301,369 | 159,335 | 9.235 |
| TCZ | 275,546 | 31,967 | 307,513 | 163,430 | 9.192 |
| TNF inhibitors | 254,346 | 26,542 | 280,889 | 152,770 | 9.433 |
| RTX | 256,066 | 66,596 | 322,662 | 167,343 | 9.214 |
| Women | | | | | |
| ABA | 273,331 | 25,859 | 299,189 | 159,135 | 9.361 |
| TCZ | 271,640 | 33,219 | 304,859 | 165,865 | 9.261 |
| TNF inhibitors | 254,067 | 27,171 | 281,238 | 153,825 | 9.537 |
| RTX | 255,880 | 72,589 | 328,469 | 171,351 | 9.274 |
| Men | | | | | |
| ABA | 259,560 | 25,553 | 285,113 | 139,152 | 9.795 |
| TCZ | 260,261 | 31,039 | 298,662 | 139,559 | 9.818 |
| TNF inhibitors | 236,423 | 26,289 | 262,712 | 132,826 | 9.990 |
| RTX | 237,216 | 61,446 | 298,662 | 146,519 | 9.789 |
| bDMARD as monotherapy at baseline | | | | | |
| ABA | 258,600 | 24,213 | 282,813 | 138,318 | 8.936 |
| TCZ | 257,374 | 30,878 | 288,252 | 146,828 | 8.774 |
| TNF inhibitors | 238,429 | 25,348 | 263,776 | 136,272 | 9.034 |
| RTX | 239,518 | 66,142 | 305,660 | 149,327 | 8.808 |
| bDMARD in combination with any csDMARD at baseline | | | | | |
| ABA | 274,680 | 26,383 | 301,064 | 161,117 | 9.704 |
| TCZ | 273,865 | 33,475 | 307,341 | 164,948 | 9.684 |
| TNF inhibitors | 255,155 | 27,556 | 282,711 | 154,118 | 9.941 |
| RTX | 256,454 | 71,314 | 327,769 | 172,290 | 9.670 |
| Methotrexate non-users at baseline | | | | | |
| ABA | 259,429 | 24,347 | 283,776 | 138,569 | 8.928 |
| TCZ | 257,861 | 30,808 | 288,669 | 146,259 | 8.784 |
| TNF inhibitors | 239,000 | 25,408 | 264,408 | 134,574 | 9.081 |
| RTX | 239,986 | 65,799 | 305,785 | 148,021 | 8.836 |
| Methotrexate users at baseline | | | | | |
| ABA | 278,476 | 26,973 | 305,449 | 166,978 | 9.897 |
| TCZ | 277,408 | 34,111 | 311,520 | 169,777 | 9.888 |
| TNF inhibitors | 258,713 | 28,133 | 286,846 | 159,872 | 10.120 |
| RTX | 260,040 | 72,685 | 332,724 | 178,868 | 9.848 |
| Primary responders | | | | | |
| ABA | 271,318 | 26,360 | 297,679 | 157,666 | 9.592 |
| TCZ | 270,042 | 33,307 | 303,349 | 161,552 | 9.564 |
| TNF inhibitors | 251,421 | 27,479 | 278,900 | 152,034 | 9.783 |
| RTX | 252,909 | 70,930 | 323,839 | 169,241 | 9.524 |
| Primary nonresponders | | | | | |
| ABA | 267,406 | 24,889 | 292,295 | 148,661 | 9.228 |
| TCZ | 266,480 | 31,524 | 298,005 | 155,930 | 9.096 |
| TNF inhibitors | 247,065 | 26,121 | 273,186 | 143,039 | 9.404 |
| RTX | 248,475 | 67,848 | 316,323 | 158,697 | 9.179 |
| Negative rheumatoid factor | | | | | |
| ABA | 329,735 | 30,761 | 360,496 | 218,678 | 12.152 |
| TCZ | 330,026 | 37,228 | 367,254 | 220,939 | 12.144 |
| TNF inhibitors | 313,626 | 32,030 | 345,656 | 211,502 | 12.370 |
| RTX | 316,079 | 74,327 | 390,405 | 241,890 | 11.960 |
| Positive rheumatoid factor | | | | | |
| ABA | 264,369 | 25,289 | 289,658 | 148,512 | 9.223 |
| TCZ | 263,413 | 32,357 | 295,770 | 154,114 | 9.159 |
| TNF inhibitors | 244,189 | 26,433 | 270,622 | 143,185 | 9.415 |
| RTX | 245,469 | 69,378 | 314,848 | 158,471 | 9.175 |

ABA = abatacept, bDMARD = biological disease-modifying anti-rheumatic drug, csDMARD, conventional synthetic disease modifying anti-rheumatic drug, ETN = etanercept, QALY = quality-adjusted life year, RA = rheumatoid arthritis, TNF inhibitor = tumor necrosis factor inhibitor, TCZ = tocilizumab
